# Assessment of flavonoids contents and in vitro antioxidant activity of Launaea procumbens

**DOI:** 10.1186/1752-153X-6-43

**Published:** 2012-05-22

**Authors:** Rahmat Ali Khan, Muhammad Rashid Khan, Sumaira Sahreen, Mushtaq Ahmed

**Affiliations:** 1 Department of Biotechnology, Faculty of Biological Sciences, University of Science and Technology, Bannu, KPK 28100, Pakistan; 2 Department of Biochemistry, Faculty of Biological Sciences, Quaid-I-Azam University Islamabad, Islamabad, Pakistan; 3 Botanical Sciences Division, Pakistan Museum of Natural History, Garden Avenue, Shakarparian, Islamabad, Pakistan

**Keywords:** *Launaea procumbens*, Scavenging of DPPH-free radicals, Superoxide radicals, HPLC, Flavonoids

## Abstract

**Background:**

*Launaea procumbens* (LP) has been used as a food supplement in Pakistan. In this study methanolic crude extract (LPME) of the whole plant and its different fractions; n-hexane (LPHE); ethyl acetate (LPEE) and chloroform (LPCE) were studied for the determination of total flavonoid and phenolics contents along with multifaceted *in vitro* scavenging assays.

**Results:**

Considerable amount of flavonoid and phenolics contents were found in all the fractions. Methanol and chloroform fraction exhibited efficient scavenging of DPPH·, ABTS·+, ·OH, superoxide, lipid peroxide and nitric oxide free radicals. Significant correlation was found between DPPH·, ABTS·+, superoxide radical, β-carotene bleaching restraint and phosphomolybdenum assay with total flavonoids and phenolics contents. High performance chromatography (HPLC) of LPME revealed the presence of vitexin, orientin, rutin, hyperoside, catechin and myricetin.

**Conclusion:**

These results reveal the presence of bioactive compounds in LPME, which might be contributed towards the various *in vitro* scavenging.

## Background

Reactive oxygen species (ROS) are generated in the normal metabolism of living organisms, and besides of their useful role in signal transduction; they are also involved in the dispersion of several degenerative diseases like malignant tumors, rheumatic joint inflammation, cataracts, Parkinson’s and Alzheimer’s disease, hypertension, diabetes, oxidative stress, tissue damages and atherosclerosis [1]. To protect the body from such effects; in addition to antioxidant enzymatic system, there are non-enzymatic biomolecules and proteins in living organisms, which act as antioxidant and free radical scavengers. However, food supplementation containing ascorbates, carotenoids, tocopherols, flavonoids and phenols play a significant role in this matter [2,3]. These bioactive natural compounds scavenge the reactive oxygen species and prevent free radicals to cause deterioration. They have the aptitude to scavenge oxygen-nitrogen derived free radicals by donating hydrogen atom or an electron, chelating metal catalysts, activating antioxidant enzymes and inhibiting oxidizes [4-6]. Based on such a type of incredible results, interest in exploration of bioactive compounds extracted from medicinal plants was increased in recent years to replace the use of synthetic drugs, which were restricted due to side effects. On the other hand, polyphenol, used as natural antioxidants, are gaining importance, due to their health benefits for humans, decreasing the risk of cardiovascular and degenerative diseases by reduction of oxidative stress and counteraction of macromolecular oxidation [7,8].

Medicinal plants are also in high demand for application of functional food or biopharmaceuticals because of consumer preferences. *Launaea procumbens* (LP) is one of the important medicinal plants widely distributed in waste places, vacant lots and in cultivated fields throughout Pakistan. Ayurvedic and herbal medicine prepared from this plant promote self healing, good health and longevity, as well as used as a food ingredient [9]. Traditionally, it has been used in the treatment of kidney disorders like painful urination, gonorrhea, and sexual diseases [10]. Chemical characterization showed that LP is composed of salicylic acid, vanillic acid, synergic acid, 2-methylresercinol and gallic acid [11]. These compounds have spasmogenic, cardiovascular, anticarcinogenic, antiinflammatory, and antioxidant properties to scavenge reactive oxygen species [12]. The present study was arranged to screen the various fractions of LP for the determination of total flavonoids and phenoilc consents, and to evaluate its antioxidant potential through scavenging of various free radicals.

## Results

### Phytochemical characterization

#### Total phenolics and flavonoids contents

Table 1 shows the presence of phenolics and flavonoids contents in various fractions of LP. The LPME possessed the highest total phenolics contents (432.8 ± 2.93) mg GAE/g while n-hexane comprised of lowest total phenolics content (188.3 ± 2.1) mg GAE/g extract. Maximum total flavonoid contents were recorded in LPME (13.98 ± 0.87) while the lowest concentration was present in LPHE (4.43 ± 0.45) mg equivalent rutin/g of dry fraction. The extraction yield of these samples varied from 4.43 ± 0.45% to be 16.28 ± 0.27% with a descending order of methanol > chloroform > ethyl acetate > n-hexane fraction. Methanol and chloroform fractions resulted in the highest amount of total extractable compounds, whereas the extraction yield with ethyl acetate and n-hexane was significantly less (*P* < 0.01) as compared to methanol and chloroform fraction.

**Table 1:** **Total phenolic content in different extracts of** ***Launaea procumbens***

**Sample**	**Total flavonoids compounds as rutin equivalent (mg/g dry extract)**	**Total phenolic compounds as mg Gallic acid equivalent (GAE mg/g extract)**	**% yield extraction**
LPME	13.98 ± 0.87^c^	432.8 ± 2.93^c^	16.28 ± 0.27^d^
LPCE	7.3 ± 0.54^b^	267.4 ± 1.3^b^	10.3 ± 0.54^c^
LPEE	8.6 ± 0.37^b^	322 ± 3.6^b^	8.6 ± 0.37b^b^
LPHE	4.43 ± 0.45^a^	188.3 ± 2.1^a^	4.43 ± 0.45^a^

Each value in the table is represented as Mean ± SD (n = 3) Means not sharing the same letter are significantly different (LSD) at P < 0.01 probability level in each column.

### HPLC quantification of flavonoids

**Figure 1:**
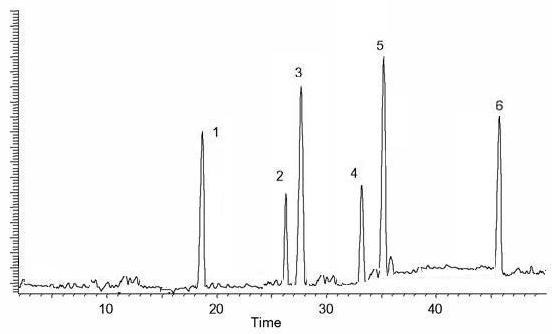
HPLC fingerprints obtained by methanolic extract of LPME Column: C18 20RBAX ECLIPSE, XDB-C18, (5 μm; 4.6 × 150 mm, Agilent USA) eluted with mixtures trifluoroacetic acid and acetonitrile indicated the presence of six compounds 1.; (kaempferol), 2.; (orientin), 3.; (rutin), 4.; (hyperuside), 5.; (quercetin) and 6.; (myricetin).

The HPLC-UV chromatogram revealed the presence of six polyphenolic compounds, including kaempferol, orientin, rutin, hyperuside, myricetin and quercetin. The investigated compounds in the methanolic extracts were quantified by integration of the peak-areas at 220 nm using an external calibration method. Calibration curves were constructed for each standard compound. Least-squares linear regression was used to determine the calibration parameters for each of standards. The linearity of all calibration curves was determined by calculating the correlation coefficients. There were some small peaks, which could not be identified; however, based on their chromatographic behaviors and UV spectra, their chemical class may correspond to unknown flavonoids compounds as presented in Figure 1. The Table 2 revealed that LPME possessed highest quantity of myricetin (1.237 ± 0.04) while hyperuside (0.335 ± 0.06) are in low concentration.

**Table 2:** **HPLC quantification of methanolic extract of** ***Launaea procumbens***

**Samples**	**Retention time**	**Concentration (μg/mg dry weight)**	**Compound**
LPME	18.5	0.607 ± 0.03	kaempferol
	26.0	0.725 ± 0.02	orientin
	27.5	0.608 ± 0.07	rutin
	33.0	0.335 ± 0.06	hyperuside
	35.0	0.897 ± 0.05	quercetin
	45.5	1.237 ± 0.04	myricetin

Mean ± SEM.

### *In vitro* antioxidant assays

#### DPPH (1, 1-diphenyl-2-picryl-hydrazyl) radical scavenging activity

**Figure 2:**
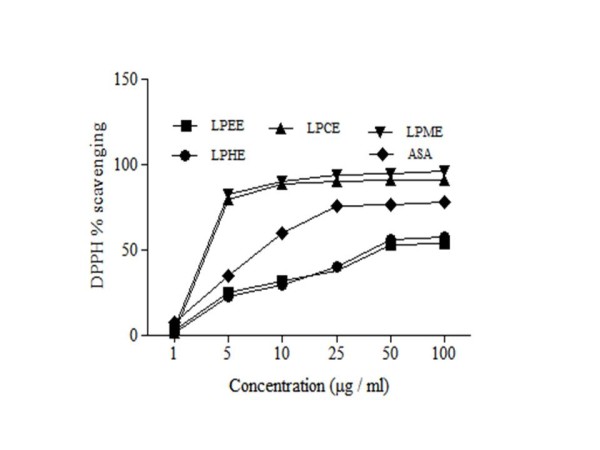
**DPPH radical scavenging activity of different extracts from the methanol extract of** ***L. procumbens*** **by different solvents at different concentrations.** Each value represents a Mean ± SD (n = 3) LPHE; LPEE; LPCE; LPME; ascorbic acid.

DPPH is a stable free radical, which has been widely used in phytomedicine for the assessment of scavenging activities of bioactive fractions. The scavenging activities of various fractions of LP extracts were determined using free radicals of 1, 1-diphenyl 1-2-picryl-hydrazyl (DPPH) (Figure 2 and Table 3). Results showed that LPME (IC50 2.6 ± 0.004 μg/ml) possessed the highest antioxidant activity as compared to other fractions while LPHE had the lowest scavenging effect (IC50 19 ± 0.04 μg/ml). The DPPH radical scavenging activities of the LPME were even less (*P* < 0.01) than those of ascorbic acid.

**Table 3:** **IC**_**50**_**of different extracts of** ***Launaea procumbens*** **for various antioxidant systems**

	**DPPH activity**	**ABTS radical Inhibition assay**	**Phosphomolybdenum assay**	**β-carotene bleachingInhibition**	**Chelating activity on Fe**^**2**^**+**	**Hydroxyl radical scavengingactivity**	**Super oxide radicalscavenging activity**	**Nitric oxide scavenging activity**	**Lipid peroxidationassay**	**Hydrogen peroxide scavenging activity**
LPME	2.6 ± 0.004^a^	50.2 ± 4.7^a^	64.27 ± 2.2^a^	51.4 ± 2.4^a^ 21^a^	63.6 ± 1.1^a^	54.2 ± 1.4^a^	70.3 ± 2.43^a^	59.4 ± 2.42^b^	60.25 ± 4.7^a^	60.4 ± 3.65^a^
LPCE	17.8 ± 0.06^d^	88.5 ± 3.8^b^	78.3 ± 2.8^b^	94.2 ± 2.6^b^	69.5 ± 3.0^a^	56.4 ± 2.0^a^	90.5 ± 3.6^b^	66.3 ± 1.6^a^	80.2 ± 4.56^b^	80.5 ± 3.0^b^
LPEE	10.4 ± 0.21^c^	104.3 ± 1.9^c^	98.3 ± 1.8^c^	125.4 ± 1. 5^c^	74.1 ± 3.06^b^	75.3 ± 2.23^b^	139.6 ± 6.3^c^	99.1 ± 3.9^b^	97.3 ± 1.5^c^	97.6 ± 2.3^c^
LPHE	19 ± 0.04^d^	122.1 ± 5.2^d^	123 ± 3.09^d^	190.21 ± 2.8^d^	92.5 ± 3.25^c^	92.5 ± 0.56^c^	220.7 ± 7.8^d^	145 ± 3.2^c^	112.2 ± 2.4^d^	100.7 ± 3.8^c^
RT	6.7 ± 0.09^b^	52.7 ± 3.2^a^	58.3 ± 1.8^a^	61.6 ± 2.4^a^	75.3 ± 3.18^b^	93.2 ± 2.6^c^	68.6 ± 2.3^a^	68.45 ± 4.2^a^	57.4 ± 3.1^a^	86.3 ± 4.0^b^
ASA	3.7 ±0.4^a^	57.4 ± 3.1^a^	72.3 ± 2.2^a^	54.7 ± 3.6^a^	65.0 ± 2.1^a^	92.5 ± 2.56^c^	70.7 ± 2.8^a^	57.2 ± 2.65^a^	52.7 ± 3.2^a^	76.3 ± 2.15^b^

Each value in the table is represented as Mean ± SD (n = 3). Means not sharing the same letter are significantly different (LSD) at P < 0.01 probability level in each column.

### ABTS radical cation assay

Scavenging capacities of various fractions of LP and ascorbic acid were assessed using ABTS (2, 2 azobis-(3-ethylbenzothiozoline-6-sulphonic acid) radical cation. Various fractions were found considerably different in their ABTS radical cation scavenging activities. The ABTS radical scavenging activity orders of various fractions of LP are; LPME (50.2 ± 1.7 μg/ml) > LPCE (88.5 ± 3.8 μg/ml) > LPEE (104.3 ± 6.9 μg/ml) > and LPHE (112.1 ± 7.2 μg/ml) respectively (Table 3). The results showed that LPME possessed significantly higher ABTS radical scavenging activity (*P <* 0.01) as compared to ascorbic acid (57.4 ± 3.1 μg/ml).

### Phosphomolybdenum assay

The basic principle to assess the antioxidant capacity through phosphomolybdenum assay includes the reduction of Mo (VI) to Mo (V) by the plant extract possessing antioxidant compounds. In the present study addition of the various fractions of LP showed that LPME (IC50 64.27 ± 2.1 μg/ml) was more effective in reduction of Mo (VI) to Mo (V) while the lowest effects were shown by LPHE (123 ± 3.09 μg/ml). The reduction of Mo (VI) to Mo (V) by administration of reference chemicals; ascorbic acid (IC50 72.3 ± 2.2 μg/ml) (Table 3), suggested the presence of effective antioxidants in various fractions of LP.

### Antioxidant activity determined by β-carotene bleaching method

The antioxidant potential of the various fractions of LP was arranged for screening through β- carotene bleaching method (Table 3). The absorbance of β-carotene was found to be decreased in the presence of 50–250 μg/ml of the various fractions or ascorbic acid. Various fractions of LP effective inhibited the oxidation of linoleic acid and subsequent bleaching of β-carotene. Among the various fractions, LPME (IC50 51.4 ± 2.4 μg/ml) showed greater inhibitory activity (*P <* 0.01) of β-carotene than other fractions and ascorbic acid (IC50 54.7 ± 3.6 μg/ml). The results suggested that various fractions possessed effective antioxidant constituents.

### Superoxide radical scavenging activity

**Figure 3:**
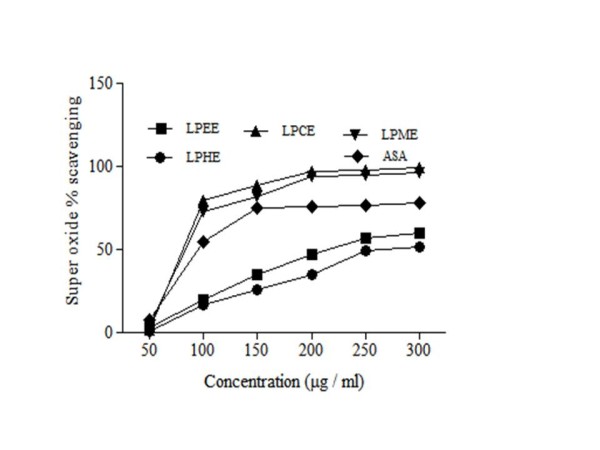
**Super oxide radical scavenging activity of different extracts from the methanol extract of** ***L. procumbens*** **by different solvents at different concentrations.** Each value represents a Mean ± SD (n = 3) LPHE; LPEE; LPCE; LPME; ascorbic acid.

Oxidation is life, but except of so many necessary processes of life, during normal metabolism of oxygen, various free radicals as well as superoxide are produced continuously. The high level of this superoxide radical is known to be harmful to cellular ingredients as, contributing to tissue damage and various diseases. The scavenging of the various fractions of LP extracts on superoxide radicals are shown in Figure 3 and Table 3. Scavenging for super oxide radicals exhibited by LPME (IC50 70.3 ± 2.43 μg/ml) was comparatively similar to ascorbic acid.

### Hydroxyl radical scavenging

Among the oxygen radicals, hydroxyl radical is the most reactive and induces severe damage to adjacent biomolecules such as protein, DNA and lipids; cause’s lipids peroxidation. Table 3 shows the hydroxyl radical scavenging of the various fractions and ascorbic acid. LPME, LPEE, LPCE and LPHE scavenged hydroxyl radicals by IC50; 51.2 ± 1.4 μg/ml, 56.4 ± 2.0 μg/ml, 75.3 ± 2.23 μg/ml and 92.5 ± 0.56 μg/ml, respectively. The scavenging affects of ascorbic acid (IC50; 92.5 ± 2.56 μg/ml) were significantly lower against the various fractions of LP.

### Hydrogen peroxide-scavenging

Hydrogen peroxide is nonreactive, but its high concentrations are toxic to living cells, and changed into free radical called hydroxyl radicals; therefore, the scavenging affects of various fractions are evaluated against this free radical (Table 3). The hydroxyl free radical in the cells can easily cross cell membranes and react with most biomolecules causes tissue damage, cancer and cell death. Thus, removal of hydroxyl free radical is necessary in to protect life. Scavenging affect of various fractions with ascorbic acid showed that LPME (IC50; 63.4 ± 3.65 μg/ml) had (*P <* 0.01) highest hydroxyl radical scavenging affect and was most potent than ascorbic acid (IC50 76.3 ± 2.15 μg/ml) respectively.

### Chelating on Fe2+

Chelation of iron plays the main role for assessing antioxidant potential of medicinal plants. The reducing power of various fractions to reduce iron ion Fe (III) into Fe (II) is shown in Table 3. Various fractions of LP showed an ability to chelate iron (II) ions in a dose-dependent manner. LPCE and LPME chelated iron ion (IC50; 69.5 ± 3.0 μg/ml; IC50; 63.6 ± 1.1 μg/ml), however, LPEE and LPHE chelate iron (II) ions (IC50; 74.1 ± 3.06 μg/ml, IC50; 92.5 ± 3.25 μg/ml), as against the iron chelating for ascorbic acid was IC50; 65.0 ± 2.1 μg/ml.

### Lipids peroxidation assay

Egg yolk lipids undergo rapid nonenzymatic peroxidation when hatched in the presence of ferrous sulfate. Lipids peroxides are likely involved in many pathological events, including inflammation, metabolic disorders, oxidative stress and cellular aging. The affects of various fractions of LP, ascorbic acids on nonenzymatic peroxidation are summarized in Table 3. The highest activity was remarked for LPME (IC50 60.25 ± 4.7 μg/ml) (*P <* 0.01) of LP and is more potent in inhibition of lipids peroxidation than other fractions. Antioxidant against lipids peroxidation was obtained for ascorbic acid (IC50 52.7 ± 3.2 μg/ml).

### Nitric oxide scavenging

Sodium nitroprusside in aqueous solution at physiological pH spontaneously produces nitric oxide, which interacts with oxygen to produce nitrite ions that can be estimated using Grries’s reagent. Scavengers of nitric oxide compete with oxygen, leading to reduced production of nitrite ions. Overall, the LPME (IC50 55.4 ± 2.42 μg/ml) showed the highest nitric oxide scavenging (*P <* 0.01) ability compared to other fractions (Table 3) and ascorbic acid. When the scavenging abilaty was expressed as Trolox equivalent, it showed the methanol extracts was more potent than the other fractions.

### Correlation with IC50 values of antioxidant and phytochemical constituents

Correlation analysis for phytochemical contents with IC50 values of radical scavenging and/or antioxidant ability of extract of LP and its various soluble fractions. The contents of phenolics and flavonoids showed significant correlation (R2 0.6721–0.998) with DPPH, superoxide, hydrogen peroxide, phosphomolybdenum and ABTS radical scavenging (Table 4) while nonsignificantly correlated with scavenging of hydroxyl and nitric oxide radicals. In addition, IC50 of chelating power of iron presented a significant correlation with flavonoids while non significant with phenolics.

**Table 4:** **Correlations between the IC**_**50**_**values of antioxidant activities, phenolics and flavonoids content of** ***Launaea procumben*** *s*

**Assays (IC**_**50**_**μg/ml)**	**Correlations R**^**2**^	
	**Phenolics**	**Flavonoids**
DPPH	0.9762^b^	0.8843^b^
Hydrogen peroxide scavenging assay	0.8101^b^	0.7657^a^
Super oxide radical scavenging	0.6987^a^	0.6765^b^
Phosphomolybdenum assay	0.7237^a^	0.7567^a^
β-carotene bleaching Inhibition	0.2003	0.2060
ABTS radical Inhibition assay	0.8821^b^	0.7797^a^
Nitric oxide scavenging activity	0.3212	0.2134
Chelating activity on Fe^2^+	0.3435	0.5564
Hydroxyl radical scavenging activity	0.3454	0.2364
Lipid peroxidation activity	0.4976	0.4123

*Launaea procumbens* methanolic extract and its soluble fractions were used in the correlation. a, b indicate significance at P < 0.05 and P < 0.01 respectively.

## Discussion

Polyphenolic flavonoids are occurring ubiquitously in food and medicinal plants. They occur as glycosides and contain several phenolics hydroxyl groups. Many flavonoids are found to be strong antioxidants effectively scavenging the reactive oxygen species because of their phenolics hydroxyl groups [13]. Our study revealed the presence of six bioactive polyphenolic flavonoids (kaempferol, orientin, rutin, hyperuside, myricetin and quercetin) in LPME, which might play an important role in improving of oxidative stress [14]. Other studies reported the presence of the bioactive constituent during chemical characterization of medicinal plants [15-18]. The data of the present study reveal the LPME contained notable amounts of phenolics compounds endowed with high antioxidant. These findings provide a good pharmacological logic for this plant in renal injuries, hormonal and sexual disorders, and antimicrobial as well as its use in folk and herbal medicine in Pakistan. It has been reported in many investigations that bioactive fractions of different medicinal plants having free radical scavenging and antioxidant, are used in many diseases like cancer, tissue inflammatory and cardiovascular disease [19-22]. Also, the number of publications on the health benefits of polyphenol has been increased [23,24]. Various free radical scavenging methods used in this study are simple and have provided reproducible results showing antioxidant properties of various fractions of LP. The antioxidant capacity of different fractions observed in this experiment could be, because of the presence of high phenolics compounds. LPME is more potent compared to other fractions and found in accordance with previous reports [25,26], which have shown that high total polyphenol content increases the antioxidant activity and proves a linear correlation between phenolics content and antioxidant activity. LPME exhibited a significant correlation as was reported by Bortolomeazzi *et al.*[27]. Phenolic compounds such as flavonoids, phenolics acid and tannins possess diverse biological activities such as anti-inflammatory, anticarcinogenic and antiatherosclerotic. The presence of these bioactive compounds might contribute to diverse scavenging effects of LP [28]. Free radicals of 1, 1-diphenyl 1-2-picrylhydrazyl (DPPH) are widely used for screening of medicinal plants to investigate their antioxidant potential. In these procedure-free, DPPH radicals when dissolves in methanol, give violet color in methanol solution. The results existed clearly indicate that in screening of various fractions of LP, methanol fraction had marked scavenging affect with IC50 2.6 ± 0.004 at 50–250 μg/ml. Our results are supported by other investigation [29]. The potential of various fractions to scavenge free radical was also assessed by their ability to quench ABTS, and depicts that LPME possessed IC50 (66.1 ± 1.02 μg/ml) value, showing the strongest activity even more than reference compounds. According to Oszmianski *et al.*[30], the antioxidant activities against ABTS or DPPH were correlated to the concentration, chemical structures, and polymerization degrees of antioxidants. Hagerman *et al.*[31] have reported that the high molecular weight phenolics (tannins) have more abilities to quench free radicals (ABTS) and their effectiveness depends on the molecular weight, the number of aromatic rings and nature of hydroxyl group’s substitution than the specific functional groups. Free radical (ABTS·+) scavenging of LP fractions might be due to the presence of high molecular weight phenolics such as catechin, and rutin derivatives in addition to other flavonoids. The phosphomolybdate method has been routinely used to evaluate the total antioxidant capacity of extracts [32]. The results showed the methanol extracts of LP (IC50 64.27 ± 2.1 μg/ml) indicated significant antioxidant activity, which was increased in a concentration-dependent manner. The results suggested that the strong antioxidant activity of extracts might be due to the presence of phenolics compounds present in the extract [33]. Recent investigation has shown that many flavonoids and related polyphenol contribute significantly to the antioxidant activity of many fruits such as red grape, vegetables and medicinal plants [34]. Methanol extracts of LP also markedly scavenge hydroxyl, hydrogen peroxide, superoxide radicals and nitric oxide as well as possesses a strong metallic reducing power, in addition to bleach β-carotene, the significant activity of LPME could be due to the presence of bioactive flavonoids. Our results agree with the results of Shukla *et al.*[35] during the screening of *in vitro* antioxidant activity and total phenolics content of ethanol leaf extract of *Stevia rebaudiana* Bert. Similar investigation was reported in other studies [36]. Oxidative stress was characterized by increased lipids peroxidation and altered nonenzymatic and enzymatic antioxidant. Cumulative evidence suggested that various enzymatic and nonenzymatic systems had been developed by mammalian cells to survive with ROS and other free radicals. Methanolic extracts of LP markedly reduced lipid peroxidation comparatively to other fractions and reference compounds. Other studies have similar contribution during characterization of lipids peroxidation. Previous studies have shown that *Mentha* extracts be able to prevent the propagation of the lipids peroxidation process in a complex lipids matrix, such as a foodstuff or biological membrane [37-39]. Flavonoids are a large group of compounds occurring ubiquitously in food plants. They occur as glycosides and contain several phenolics hydroxyl groups in their ring structure, capable of antioxidant activities [13]. In our study, flavonoids showed a concentration dependent antioxidant activity of different fractions of LP. Phenols are secondary metabolites in plants and are known to possess a wide range of therapeutic uses, such as antioxidant, antimutagenic, anticarcinogenic, free radical-scavenging and also decrease cardiovascular complications [40]. The scavenging ability of the phenols is mainly, because of the presence of hydroxyl groups. From the results obtained, it is inferred that total phenol contents were present in the reasonable amount in LPME and its derived fractions. A previous report also supports our results [41].

## Materials and methods

### Chemicals

Nitroblue tetrazolium (NBT), β-nicotinamide adenine dinucleotide reduced (β-NADH), 2-deoxy- D-ribose, linoleic acid, ammonium thiocyanate, β-carotene, 3-(2-pyridyl)-5, 6 bis (4-phenylsulfonic acid)-1,2,4-triazine (ferrozine), Phenazine methosulphate (PMS), 2,2-diphenyl-1-picrylhydrazyl (DPPH), ethylenediamine tetra acetic acid (EDTA), rutin, ascorbic acid, gallic acid, potassium ferricyanide; trichloroacetic acid (TCA), thiobarbituric acids (TBA) were obtained from Sigma Aldrich Chemical Co. (USA). All other reagents were of analytical grade.

### Plant collection

Plants of LP at maturity were collected from Wah Cantt, city Rawalpindi (Pakistan). Plants were identified and a specimen was submitted at Herbarium of Pakistan, Quaid-I-Azam University Islamabad, Pakistan. Whole plant (leaves, stem, flowers and seeds) were shades dried at room temperature for two weeks, chopped, ground mechanically of mesh size 1 mm.

### Preparation of plant extracts

Five kg powder of *Launaea procumbens* was extracted twice in 10 liter of methanol with random shaking, after a week the extract was filtered through whatmann filter paper No. 45, filtrate was mixed and evaporated through rotary vacuum evaporator at 40°C to get 362 g methanolic crude extracts (LPME). The crude extract was suspended in water and fractionated by liquid: liquid partition with solvents of increasing polarity; starting from n-hexane (23 g; LPHE), ethyl acetate (43 g; LPEE) and chloroform (67 g; LPCE). All the fractions were stored at four °C for further phytochemical and *in vitro* investigations.

### Phytochemicals characterization

#### Determination of the total phenolics contents

Total phenolics contents (TPC) were estimated using the method of Singleton and Rossi [42]. Two hundred micro liters (1–5 mg/ml; dissolved in respective solvent) of each fraction was added in ten milliliter of 1:10 folin-ciocalteu reagent and incubated for 5 min before the addition of 7 ml of 0.115 mg/ml Na2CO3. The resulting solution was incubated a further 2 h before absorbance readings were taken at 765 nm. Gallic acid was used in the calibration curve. Results were expressed as mg gallic acid (GAE)/g dried plant extract. Data for each fraction was recorded in triplicate.

### Determination of the total flavonoids

Total flavonoids content was determined by using a method described by Sakanaka *et al.,*[43]. Briefly, 0.25 ml of each fraction (1–5 mg/ml; dissolved in respective solvent) and rutin standard solution (15–250 μg/ml) was mixed with 1.25 ml of distilled water in a test tube, followed by addition of 75 μl of a 5% (w/v) sodium nitrite solution. After 6 min, 150 μl of 10% (w/v) aluminum chloride solution was added, and the mixture was allowed to stand for a further 5 min before 0.5 ml of 1 M NaOH was added. The mixture was made up to 2.5 ml with distilled water and mixed well. The absorbance was measured immediately at 510 nm. The results of samples were expressed as mg of rutin equivalents of total dried fractions. All fractions were run in triplicate.

### High performance liquid chromatography’

One gram powder was extracted with 6 ml of 25% hydrochloric acid and 20 ml methanol for 1 h.

The obtained extract was filtered to a volumetric flask. The residue was heated twice with 20 ml of methanol for 20 min. The combined extract was diluted with methanol to 100 ml. 5 ml portion of the solution was filtered and transferred to a volumetric flask and diluted with 10 ml of methanol. The sample (10 μl) was injected into the HPLC apparatus. Samples were analyzed on Agilent HPLC. Separation was carried out through column (5 μm; 4.6 × 150 mm, Agilent) with UV–vis detector. Solvent A (0.05% trifluoroacetic acid) and solvent B (0.038%trifluoroacetic acid in 83% acetonitrile (v/v) with the following gradient: 0–5 min, 15% B in A, 5–10 min, 70% B in A, 10–15 min, 70% B in A are used for separation. The flow rate was 1 ml/min and injection volume was 10 μl. Six different standards compounds (myricetin, catechin, vitexin, orientin, hyperuside, and rutin) were run for comparable detection and optimized. The calibration curves were defined for each compound in the range of sample quantity 0.02–0.5 μg. All samples were assayed in triplicate. All quantitative data were explained by analyst software.

### Antioxidant assays

#### DPPH radical scavenging

The free-radical scavenging activity was measured by using 1, 1-diphenyl-2-picryl-hydrazyl (DPPH) assay. DPPH assay was performed according to the procedure as reported by Gyamfi *et al.*[44]. DPPH solution was prepared by dissolving 3.2 mg in 100 ml of 82% methanol. 2.8 ml of DPPH solution was added to glass vial followed by the addition of 0.2 ml of test sample solution, in methanol, leading to the final concentration of 1 μg/ml, 5 μg/ml, 10 μg/ml, 25 μg/ml, 50 μg/ml and 100 μg/ml. Mixture of DPPH, and each fraction was shaken well and kept in the dark at controlled room temperature (25–28°C) for 1 h. After incubation change in color was measured at 517 nm. Mixture of 2.8 ml of 82% methanol and 0.2 ml of methanol were used as blank while 0.2 ml of methanol and 2.8 ml of DPPH solution were taken as control. The test of each fraction was performed in triplicate. Percentage inhibition was measured according to following formula and IC50 value was calculated by graph pad prism software.

(1) % scavenging=Abs. of control–Abs. of fraction×100/ Abs. of control

### ABTS radical cation assay

ABTS radical cation assay was carried out using the protocol of Re *et al.*[45]. According to this protocol, ABTS (2, 20-azinobis-(3-ethylbenzothiazoneline-6-sulphonic acid, 7.4 mM) used as the free-radical provider, was treated with potassium persulfate (2.45 mM) to produce free radicals. The solution was diluted to obtain an absorbance of 1.5–2.5 at 414 nm with 98% of ethanol, before used. Reagent (3 ml) was transferred to the glass cuvettes with one of them containing 3 ml ethanol as blank. The initial absorbance of the reagents in the glass cuvettes was recorded at 414 nm. 100 μl of each fraction (0.05–0.250 mg/ml) were transferred into the cuvettes containing the reagent, and the mixtures were shaken thoroughly. The mixture in the cuvette was examined after 90 min using a UV–vis spectrophotometer. Antioxidant capacity of the ascorbic acid was also determined. The capability to scavenging the ABTS radical cation was calculated using the following equation;

(2) $$\%\text{ ABTS radical cation scavenging ability}=\left(\text{A}1-\text{A}2/\text{A}1\right)\times 100$$

Where A1 is the absorbance of the control (ABTS solution without test sample), and A2 is the absorbance in the presence of the test sample. The results reported are expressed as their IC50 through Graph prism pad software.

### Phosphomolybdenum assay

The antioxidant activity of fractions was evaluated by phosphomolybdenum method according to the procedure of Prieto *et al.*[32]. An aliquot of 0.1 ml of each fraction (dissolved in respective solvent) was combined in a vial with 1 ml of reagent solution (0.6 M sulphuric acid, 28 mM sodium phosphate and 4 mM ammonium molybdate). The vial was capped and incubated in a water bath at 95°C for 90 min. After the incubation, samples were cooled to room temperature, and the absorbance of the mixture was measured at 765 nm against a blank. Percent inhibition was calculated by the following formula while IC50 was calculated through Graph prism pad software.

(3) $$\%\;\text{inhibition}=\left(1–\text{absorbance of sample}/\text{absorbance of control}\right)\times 100$$

### Antioxidant activity by β-carotene bleaching method

The antioxidant activity of each fraction was evaluated using the ß-carotene-linoleate model system, as described by Sun and Ho [46]. 2 mg of β-carotene were dissolved in 10 ml chloroform and 1 ml β-carotene solution was mixed with 20 mg of purified linoleic acid and 200 mg of Tween 40 emulsifiers. Chloroform was then evaporated under a gentle stream of nitrogen, and the resulting mixture was immediately diluted with 50 ml of distilled water. To an aliquot of 5 ml of this emulsion, 0.2 ml of each extract (0.05–0.250 mg/ml) or ascorbic acids were added and mixed well. The absorbance at 470 nm, which was regarded as t0, was measured, immediately, against a blank consisting of the emulsion without β-carotene. The capped tubes were placed in a water bath at 50°C, and the absorbance was measured after every 15 min up to 120 min. For the positive control, sample was replaced with ascorbic acid. A negative control consisted of 0.2 ml of distilled water or solvent instead of extract or reference antioxidant was used. All samples were assayed in triplicate. The antioxidant activity (AA) was measured in terms of successful bleaching of β-carotene by using the following equation; $\text{AA}=(\left(1–\left(\text{A}0–\text{At}/\text{A}0–\text{A}0\text{t}\right)\right)\times 100$

Where A0 and A0 are the absorbance values measured at zero times during the incubation for each fraction and control, respectively. At an A0t was the absorbance values measured for each fraction and control, respectively, after incubation for 120 min. The results were expressed as IC50.

### Superoxide radical scavenging activity

Superoxide radical scavenging activity of each fraction was determined by the nitroblue tetrazolium reduction method [47]. One milliliter of nitroblue tetrazolium (NBT) solution (l M NBT in 100 mM phosphate buffer, pH 7.4), 1 ml NADH solution (l M NADH in 100 mM phosphate buffer, pH 7.4) and 0.1 ml of the extracts (0.50–0.250 mg/ml) and ascorbic acid (0.050–0.250 mg/ml) were mixed. The reaction was started by adding 100 μl of PMS solution (60 μM PMS in 100 mM phosphate buffer, pH 7.4) to the mixture. The reaction mixture was incubated at 25°C for 5 min, and the absorbance at 560 nm was measured against blank samples, containing all the reagents except the PMS. The positive and negative controls were subjected to the same procedures as the sample, except that for the negative control, only the solvent was added, and for the positive control, sample was replaced with ascorbic acid. All measurements were made in triplicate. The abilities to scavenge the superoxide radical were calculated using the following equation;

(5) $$\begin{array}{l} \%\text{ superoxide radical scavenging activity}=\\ \left(1-\text{absorbance of sample at }560\text{nm}/\text{absorbance of control at }560\text{nm}\right)\times 100 \end{array}$$

IC50 was calculated through software.

### Hydroxyl radical scavenging activity

The effect of extracts on hydroxyl radicals was assayed by using the deoxyribose method [48]. Solution of each fraction and ascorbic acid (ASA) was prepared in methanol. The reaction mixture contained; 450 μl of 0.2 M sodium phosphate buffer (pH 7.0), 150 μl of 10 mM 2- deoxyribose, 150 μl of 10 mM FeSO_4_-EDTA, 150 μl of 10 mM H_2_O_2_, 525 μl of H_2_O, and 75 μl of sample solution (0.050–0.250 mg/ml). The reaction was started by the addition of H_2_O_2_. After incubation at 37°C for 4 h, the reaction was stopped by adding 750 μl of 2.8% trichloroacetic acid and 750 μl of 1% TBA in 50 mM NaOH, the solution was boiled for 10 min, and then cooled in water. The absorbance of the solution was measured at 520 nm. Ascorbic acid (0.05–0.250 mg/ml) was used as positive controls. The ability to scavenge the hydroxyl radical was calculated using the following equation;

(6) $$\%\text{ superoxide radical scavenging activity}=\left(1-\text{absorbance of sample}/\text{absorbance of control}\right)\times 100$$

### Hydrogen peroxide-scavenging activity

The ability of the extracts to scavenge hydrogen peroxide was determined according to the method of Ruch *et al.*[49]. A solution of hydrogen peroxide (2 mM) was prepared in 50 mM phosphate buffer (pH 7.4). Hydrogen peroxide concentration was determined spectrophotometrically at 230 nm absorption, using the molar extinction coefficient for H_2_O_2_ of 81 mol^-1^ cm^-1^. Samples of various fractions (0.050–0.250 mg/ml) and ascorbic acid (0.05–0.250 mg/ml) were transferred into the test tubes, and their volumes were made up to 0.4 ml with 50 mM phosphate buffer (pH 7.4). After addition of 0.6 ml hydrogen peroxide solution, tubes were vortex and absorbance of the hydrogen peroxide at 230 nm was determined after 10 min, against a blank. 50 mM phosphate buffer without hydrogen peroxide was used as blank. Hydrogen per oxide scavenging ability was calculated by following equation:

(7) $$\text{Hydrogen peroxide scavenging activity}=\left(1–\text{absorbance of sample}/\text{absorbance of control}\right)\times 100$$

IC50 was calculated through graph prism pad software.

### Chelating activity on Fe2+

The extracts were assessed for their ability to compete with ferrozine for iron (II) ions in free solution. The chelating ability of ferrous ions by various fractions was estimated by the method of Dinis *et al.*[50]. Extracts (0.05–250 mg/ml), 2.5 ml were added to a solution of 2 mM FeCl_2_.4H_2_O (0.05 ml). The reaction was initiated by the addition of 5 mM ferrozine (0.2 ml); the mixture was shaken vigorously and left standing at room temperature for 10 min. Absorbance of the solution was then measured at 562 nm against the blank performed in the same way using FeCl_2_ and water. EDTA (0.625–5 μg/ml) served as the positive control, and a sample without extract or EDTA served as the negative control. All tests were run in triplicate and averaged. The percentage of inhibition of ferrozine-Fe^2^ + complex formation was calculated using the formula:

(8) $$\text{Chelating activity }\%=\left(1–\text{absorbance of sample}/\text{absorbance of control}\right)\times 100$$

### Lipid peroxidation assay

Lipid peroxidation assay was performed according to modified protocol of Banerjee *et al.*[51] to measure the lipid peroxide formed, using egg yolk homogenates as lipid-rich media [52]. Egg homogenate (0.5 ml of 10%, v/v) and 0.1 ml of each fraction and ascorbic acid (0.5–0.250 mg/ml) was dissolved in respective solvent; were added to a test tube and made up to 1 ml with distilled water. 0.05 ml of FeSO_4_ (0.07 M) was added to induce lipid peroxidation and incubated for 30 min. Then 1.5 ml of 3.5 M acetic acid (pH adjusted to 3.5 with NaOH) and 1.5 ml of 0.06 M TBA in 0.04 M sodium dodecyl sulphate and 0.05 ml of 1.2 M of TCA was added, and the resulting mixture was vortex and then heated at 95°C for 60 min. To eliminate this non-MDA interference, another set of samples was treated in the same way, incubating without TBA, to subtract the absorbance for fraction and reference compounds. After cooling, 5 ml of butan-1-ol was added to each tube and centrifuged at 3000 × g for 10 min. The absorbance of the organic upper layer was measured at 532 nm. Inhibition of lipid peroxidation (%) by the sample was calculated according to the following formula:

(9) $$\%\text{inhibition}=\left(1-\text{E}/\text{C}\right)\times 100$$

Where C is the absorbance value of the fully oxidized control, and E is {(A532 + TBA)–(A532– TBA)}.

### Nitric oxide scavenging activity

The nitric oxide scavenging activity was conducted based upon the method by Rai *et al.*[53]. 0.5 ml of 10 mM sodium nitroprusside in phosphate buffered-saline was mixed with 0.5 ml of different concentrations of the various fractions and control and incubated in the dark at room temperature for 150 min. After the incubation period, 1 ml of sulfanilic acid reagent (0.33% sulfanilic acid in 20% glacial acetic acid) was added to 0.5 ml of the reaction mixture. After 5 min incubation, 1 ml of 0.1% naphthyl ethylene diamine dihydrochloride was added and incubated for 30 min at 25°C. The absorbance of the chromophore formed was read at 540 nm. Ascorbic acid was used as positive control and results were expressed as percentage inhibition of nitric oxide. The nitric oxide scavenging activity of the extracts was also measured using the Trolox standard curve and results were expressed as mM Trolox equivalent antioxidant capacity (TEAC) per g dried fraction. All determinations were performed in triplicates.

### Statistical analysis

All assays were carried out in triplicates, and results are expressed as mean ± SD. ANOVA test was used to analyze the differences among IC50 of various fractions for different antioxidant assays, with least significance difference (LSD) *P* < 0.01 as a level of significance. Experimental results were further analyzed for Pearson’s correlation coefficient of phenolics, flavonoids with different antioxidant assays and tested for significance by student’s test (*P* < 0.05; *P* < 0.01). The IC50 values were calculated using graph pad prism software.

## Conclusion

The results obtained in this study have considerable value with respect to the antioxidant activities of LPME. The presence of these activities is attributed to the phenolics and poly phenolics compounds such as myricetin, catechin, vitexin, orientin, hyperoside, and rutin, revealed in HPLC. Our results suggested that the extract can be utilized as an effective and safe antioxidant source, as ethnomedicine and on a commercial basis for the development of drugs.

## Competing interest

The authors declare that they have no competing interests.

## Authors’ contributions

RAK made a significant contribution to acquisition of data, analysis, drafting of the manuscript. MRK and SS has made a substantial contribution to conception and design, interpretation of data, drafting and revising the manuscript for intellectual content. All authors read and approved the final manuscript.
